# 3C-digital PCR for quantification of chromatin interactions

**DOI:** 10.1186/s12867-016-0076-6

**Published:** 2016-12-06

**Authors:** Meijun Du, Liang Wang

**Affiliations:** Department of Pathology and MCW Cancer Center, Medical College of Wisconsin, Milwaukee, WI 53226 USA

**Keywords:** Chromatin interaction, Chromosome conformation capture, Digital PCR, Quantitative PCR

## Abstract

**Background:**

Chromosome conformation capture (3C) is a powerful and widely used technique for detecting the physical interactions between chromatin regions in vivo. The principle of 3C is to convert physical chromatin interactions into specific DNA ligation products, which are then detected by quantitative polymerase chain reaction (qPCR). However, 3C-qPCR assays are often complicated by the necessity of normalization controls to correct for amplification biases. In addition, qPCR is often limited to a certain cycle number, making it difficult to detect fragment ligations with low frequency. Recently, digital PCR (dPCR) technology has become available, which allows for highly sensitive nucleic acid quantification. Main advantage of dPCR is its high precision of absolute nucleic acid quantification without requirement of normalization controls.

**Results:**

To demonstrate the utility of dPCR in quantifying chromatin interactions, we examined two prostate cancer risk loci at 8q24 and 2p11.2 for their interaction target genes *MYC* and *CAPG* in LNCaP cell line. We designed anchor and testing primers at known regulatory element fragments and target gene regions, respectively. dPCR results showed that interaction frequency between the regulatory element and *MYC* gene promoter was 0.7 (95% CI 0.40–1.10) copies per 1000 genome copies while other regions showed relatively low ligation frequencies. The dPCR results also showed that the ligation frequencies between the regulatory element and two *Eco*RI fragments containing *CAPG* gene promoter were 1.9 copies (95% CI 1.41–2.47) and 1.3 copies per 1000 genome copies (95% CI 0.76–1.92), respectively, while the interaction signals were reduced on either side of the promoter region of *CAPG* gene. Additionally, we observed comparable results from 3C-dPCR and 3C-qPCR at 2p11.2 in another cell line (DU145).

**Conclusions:**

Compared to traditional 3C-qPCR, our results show that 3C-dPCR is much simpler and more sensitive to detect weak chromatin interactions. It may eliminate multiple and complex normalization controls and provide accurate calculation of proximity-based fragment ligation frequency. Therefore, we recommend 3C-dPCR as a preferred method for sensitive detection of low frequency chromatin interactions.

**Electronic supplementary material:**

The online version of this article (doi:10.1186/s12867-016-0076-6) contains supplementary material, which is available to authorized users.

## Background

Chromosome conformation capture (3C) has been widely used for detecting the physical interactions of chromosomal regions in vivo [1]. In general, 3C library is first built by three basic steps involving fixation of chromatin spatial configuration by formaldehyde, digestion of cross-linked chromatin with restriction enzymes, and intra-molecular ligation of digested fragments that favors proximity. Chromatin interactions are then detected by measuring ligation frequency of two interacting fragments by polymerase chain reaction (PCR) [2–4]. Initial 3C assays estimate ligation frequency based on intensity of ethidium bromide-stained PCR products separated by agarose gel electrophoresis [3]. The gel-based assays, however, are hardly quantitative, making it difficult to differentiate subtle difference or detect weak signals. With advent of real-time quantitative PCR (qPCR), quantification of 3C ligation frequency becomes more accurate by monitoring the signal strength after each amplification cycle [4]. Due to relatively low ligation frequency in 3C library [1, 5], the qPCR assay usually detects amplification signals at high cycle threshold (Ct) (such as Ct ≥ 35), which significantly reduces the assay’s sensitivity. In addition, current 3C-qPCR is complex because randomly ligated control is needed to normalize the amplification efficiency of different primer pairs.

Recently, digital PCR (dPCR) has been emerged as a powerful tool for nucleic acid quantification, in particular, for rare molecule detection [6]. The technology detects number of targeted nucleic acids for absolute quantification by molecular counting. During dPCR, DNA samples are partitioned into thousands or millions of individual PCR reactions. Due to significant dilution, each reaction partition contains zero or one target molecule, sometimes multiple copies if dilution is not sufficient. After PCR amplification, each independent reaction can be defined as positive or negative for the target molecule by intensity of its recorded fluorescence signal [6]. Characterized by high sensitivity and specificity, the dPCR is increasingly being used for various applications such as absolute nucleic acid quantification, rare mutation detection, and copy number variation [7–9]. Here we reported a 3C-dPCR assay by incorporating dPCR technology into 3C assay [10]. We tested this assay at two prostate cancer risk regions of 8q24 and 2p11.2 for their interaction target genes *MYC* and *CAPG* [11, 12]. Our results show that 3C-dPCR is easier to use and more sensitive in determining chromatin interactions. The 3C-dPCR is likely to offer a valuable alternative method for accurate quantification of low frequency chromatin interactions.

## Result

### 3C-dPCR workflow

**Fig. 1:**
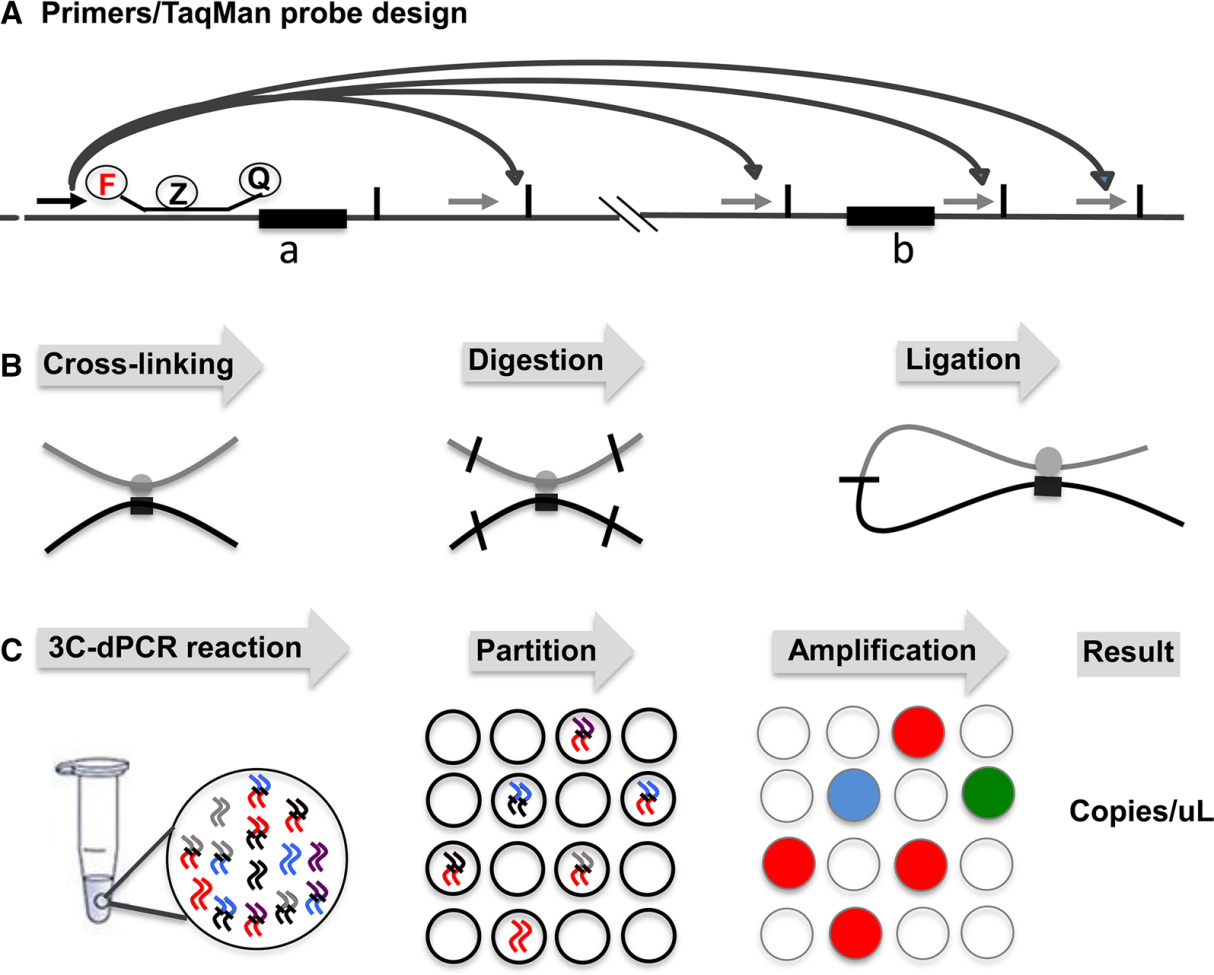
3C-dPCR workflow. **A** TaqMan probe and primer design. The locations of the two possible interaction fragments (*a* and *b*) are shown (*black rectangle*). Restriction sites used in the 3C assay are depicted as *small vertical bars* in *black*. The relative positions of anchor primer (*black arrow*), the TaqMan probe (F-Z-Q) and test primers (*grey arrows*) are also depicted. *F* fluorophore, *Z* internal quencher, *Q* quencher. **B** Three essential steps of 3C assays: 1. Interacting chromatin segments are cross-linked by formaldehyde. 2. Cross-linked chromatins are digested by a selected restriction enzyme. 3. Cross-linked fragments undergo intra-molecular ligation. **C** Principle of 3C-dPCR. The reaction mixture containing 3C DNA is prepared and partitioned into thousands of reaction wells. Due to significant dilution, each reaction well receives 0–1 target ligation products. After PCR amplification, the fluorescence signals are imaged and copy numbers of target ligations are reported as copies/μL. In the 3C-dPCR reaction and partition steps, curved lines in *blue*, *red*, *black*, *grey* and *purple curve* in the *circle* represent the different DNA molecules, including ligation products in 3C libraries. In the amplification step, the *blue dot* (well) shows target amplification signal; the *red dots* (wells) indicate the genome copy number signal; the *green dot* (well) displays the overlap of target and genome copy number signals

To identify a chromatin interaction through looping structure, it is necessary to show that the two interaction fragments have higher contact frequency than randomly ligated fragments. The first step of the procedure is to design primer and probe. In principle, a series of primers covering both pre-defined regions should be selected. In this study, we examined one fixed anchor primer (interaction hot spot) in combination with a series of test primers (covering target region). TaqMan probe was located downstream of the anchor primer (Fig. 1A). The second step is to build a 3C library including chromatin crosslinking, restriction enzyme digestion and intra-molecular fragment ligation (Fig. 1B). The third step is to measure interaction (ligation) frequency using primers specific for the restriction fragments of interest. After PCR amplification in a digital PCR system, positive and negative reactions were determined by the fluorescence signal intensity. The number of the concentration of ligation product was reported as copies/μL (Fig. 1C).

### Characterization of dPCR for detection of 3C product

**Fig. 2:**
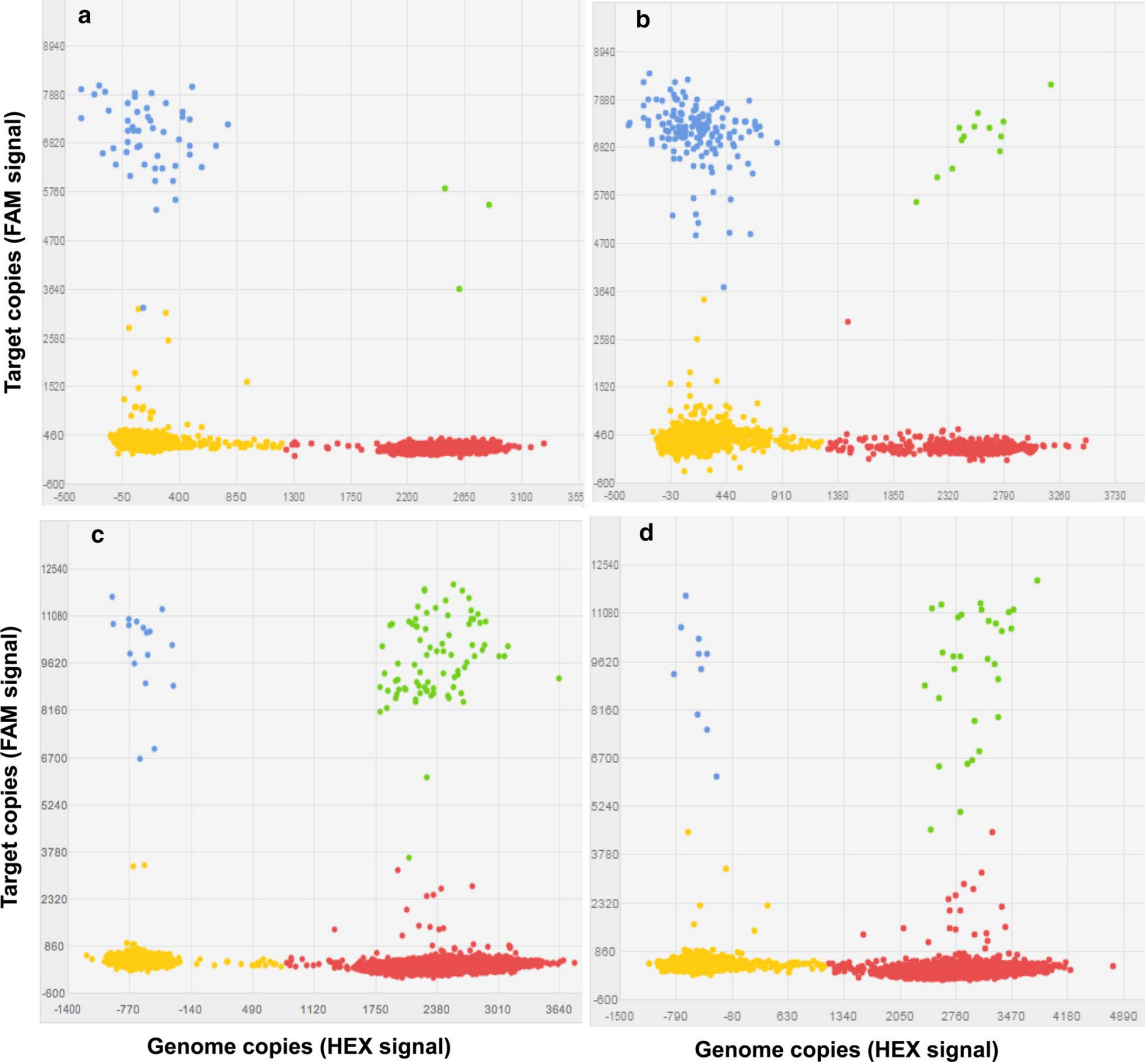
Representative duplex 3C-dPCR plots in cell line DU-145. **a** *Eco*RI digestion efficiency test, 197 copies of undigested *Eco*RI fragment per 1000 genome using a primer pair across the *Eco*RI site 2L at 2q11.2 loci. **b** Strong interaction signal for self-ligation fragment by the primer designed on the same fragment with anchor primer and paired with the anchor primer at 2q11.2, 44 copies per 1000 genome. **c** Moderate interaction signals for nearby ligation by the adjacent primer designed on the fragment next to the anchor primer at 2q11.2, 3.6 copies per 1000 genome. **d** Relative weak interaction signal for long-distance interaction between 2L with *Eco*RI fragment T7 covering the promoter of gene *CAPG*, 1.7 copies per 1000 genome. The x-axis displays the amplitude of genome copy number control (labeled by HEX, *red*) and the y- axis is signal strength of target ligation products (labeled by FAM, *blue*). The signals in the *lower left quadrant* are negative for both targets (*yellow*). The signals in the *upper right quadrant* are positive for both targets (*green*)

dPCR assay provides a convenient and straightforward approach to run up to millions of PCR reactions in parallel. In this study, we applied 3D Digital PCR system and performed duplex dPCR by including both target and genome copy control. Figure 2a and b displayed the representative plot showing digestion efficiency and self-ligation rate, where we observed 197 copies undigested *Eco*RI fragments and 44 self-ligated copies per 1000 genome copies. Figure 2c indicated moderate adjacent fragment ligation with 3.6 copies per 1000 genome copies. Figure 2d showed the representative plot of long-range chromatin interaction with 1.7 copies per 1000 genome copies. For each plot, signals in the lower left quadrant were negative (yellow) for both targets, in the lower right quadrant were positive for genome copy number (red), and in the upper left quadrant were positive for target fragment ligation (blue). The green signals between red and blue were positive for both the target and genome copy control. The intensity of fluorescence signals reflected target copy numbers after PCR amplification. The signals were specific to each primer/probe set.

### Detection of chromatin interactions at selected target regions

**Fig. 3:**
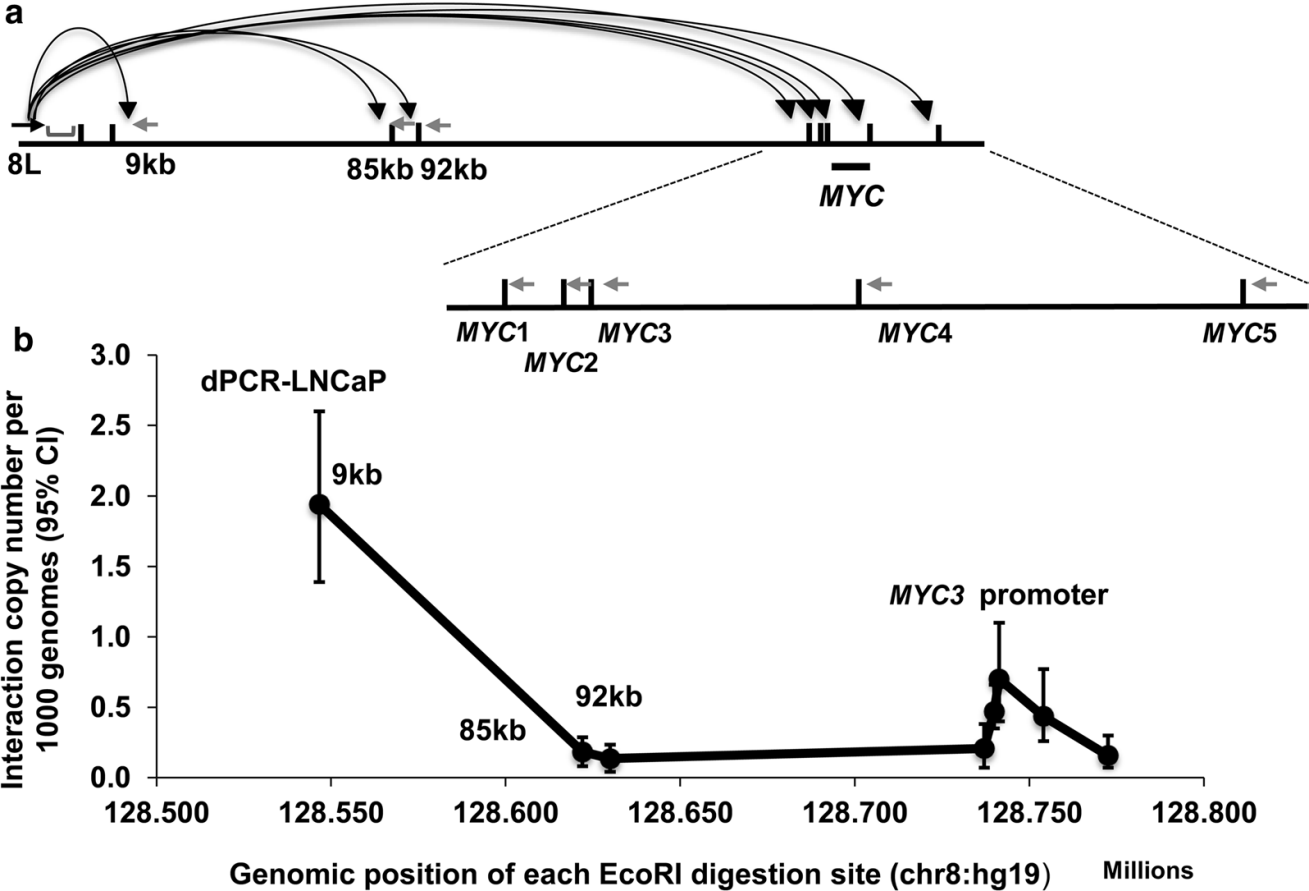
Interactions between prostate cancer risk region 1 and *MYC* gene locus at 8q24. **a** The anchor primer 8L, TaqMan probe; five target test primers (*MYC*1–*MYC*5) and three control test primers (9, 85, and 92 kb) are designed for the detection of the *cis*-acting interactions. *Small vertical bars* in *black* represent *Eco*RI digestion sites. *Black* and *grey arrows* show the anchor primer and test primers, respectively. The TaqMan probe is depicted as *grey bar*. **b** The copy number of ligation products at each selected restriction site. The highest interaction is at *MYC*-3 fragment, which contains the *MYC* promoter region. The y-axis displays the ligation copy numbers at each *Eco*RI site per 1000 genome. The x-axis is the genomic position of each *Eco*RI site. The *error bars* represent 95% CIs

To determine the ligation frequency between the two restriction fragments in 3C libraries, we first tested a previous reported interaction between 8q24 region 1 and *MYC* gene [11], we examined an anchor primer 8L at *Eco*RI site (chr8: 128537495) paired with five test primers at *MYC* gene locus (*MYC*1–5). The anchor primer was also paired with three other test primers at 9, 85 and 92 kb downstream as nearby ligation and random ligation controls (Fig. 3a). The highest ligation frequency (approximately 2%) was observed for the fragment located directly upstream of the anchor fragment (Fig. 3b). The interaction frequency between the regulatory element and *MYC* gene promoter fragment (*MYC*3), ~200 kb away from the anchor fragment, was 0.7 copies (95% CI 0.40–1.10) per 1000 genome copies while the regions (−85 and −92 kb regions) assumed to be looped out from the hub [11] showed ligation frequencies of 0.18 (95% CI 0.08–0.29) and 0.16 (95% CI 0.07–0.30), respectively. Moreover, there are no obvious signals for ddH_2_O and random ligated genomic DNA negative control (data not shown).

**Fig. 4:**
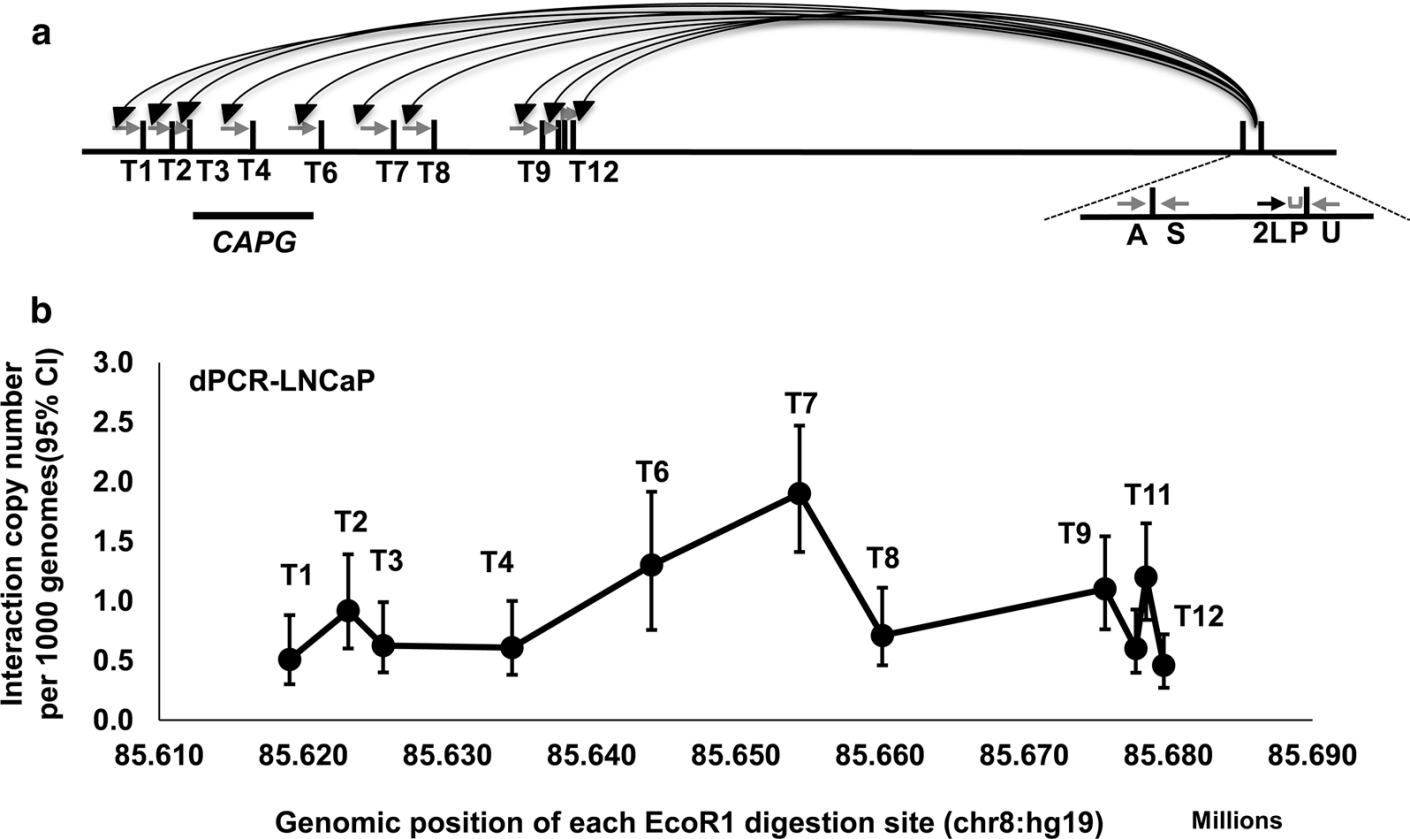
Interactions between 2L and the cluster of* Eco*RI fragments on *CAPG* gene locus at 2p11.2. **a** Anchor primer 2L, TaqMan probe on prostate cancer SNP risk region and eleven test primers (from T1 to T12) around the *CAPG* gene locus are selected for the detection of the long-range interactions. *Small vertical bars* in *black* represent *Eco*RI digestion sites. *Black* and *grey arrows* show the anchor primer and test primers, respectively. The TaqMan probe is depicted as *grey bar*. **b** The copy number of ligation products at each selected restriction site. Relative strong interaction signals were found with the *Eco*RI fragments covering primer T6, T7 and T11. The y-axis displays the ligation copy numbers at each *Eco*RI site per 1000 genome. The x-axis is the genomic position of each *Eco*RI site. *Error bars* represent standard deviation of triplicate dPCR results. *A* adjacent ligation primer, *S* self-ligation primer, *U* undigested control primer, *P* TaqMan probe. The *error bars* represent 95% CIs

We further tested the ligation frequency between prostate cancer risk locus 2p11.2 and gene *CAPG,* which was described in our previous study [12]. The anchor primer at 2q11.3 was designed at the position (chr2: 85778503) named 2L. Eleven test primers were designed on 2q11.3 covering twelve *Eco*RI cutting sites from chr2: 85619044 (T1) to chr2: 85679686 (T12), which corresponded to the promoter and nearby region of *CAPG* (Fig. 4a). We observed strong interaction signals at *Eco*RI fragments containing primer T7 and T6 with the ligation frequency 1.9 copies (95% CI 1.41–2.47) and 1.3 copies (95% CI 0.76–1.92) per 1000 genome copies, respectively. The interaction signals were reduced on either side of this *Eco*RI site. Another interaction peak was with primer T11, the interaction frequency was 1.2 copies (95% CI 0.84–1.65) molecule per 1000 genomes. The lowest interaction signal was 0.45 copies (95% CI 0.27–0.72) per 1000 genomes (Fig. 4b). We also examined the frequency of self-ligation in the 3C library by pairing the anchor primer with a primer on the same fragment. A primer pair across the *Eco*RI site was used to test the enzyme digestion efficiency. We found 44 copies (95% CI 34–58) per 1000 genomes for the frequency of self-ligation and 197 copies (95% CI 172–226) per 1000 genomes for the undigested *Eco*RI fragments (Fig. 2a, b).

### Comparison of 3C-dPCR with 3C-qPCR

**Fig. 5:**
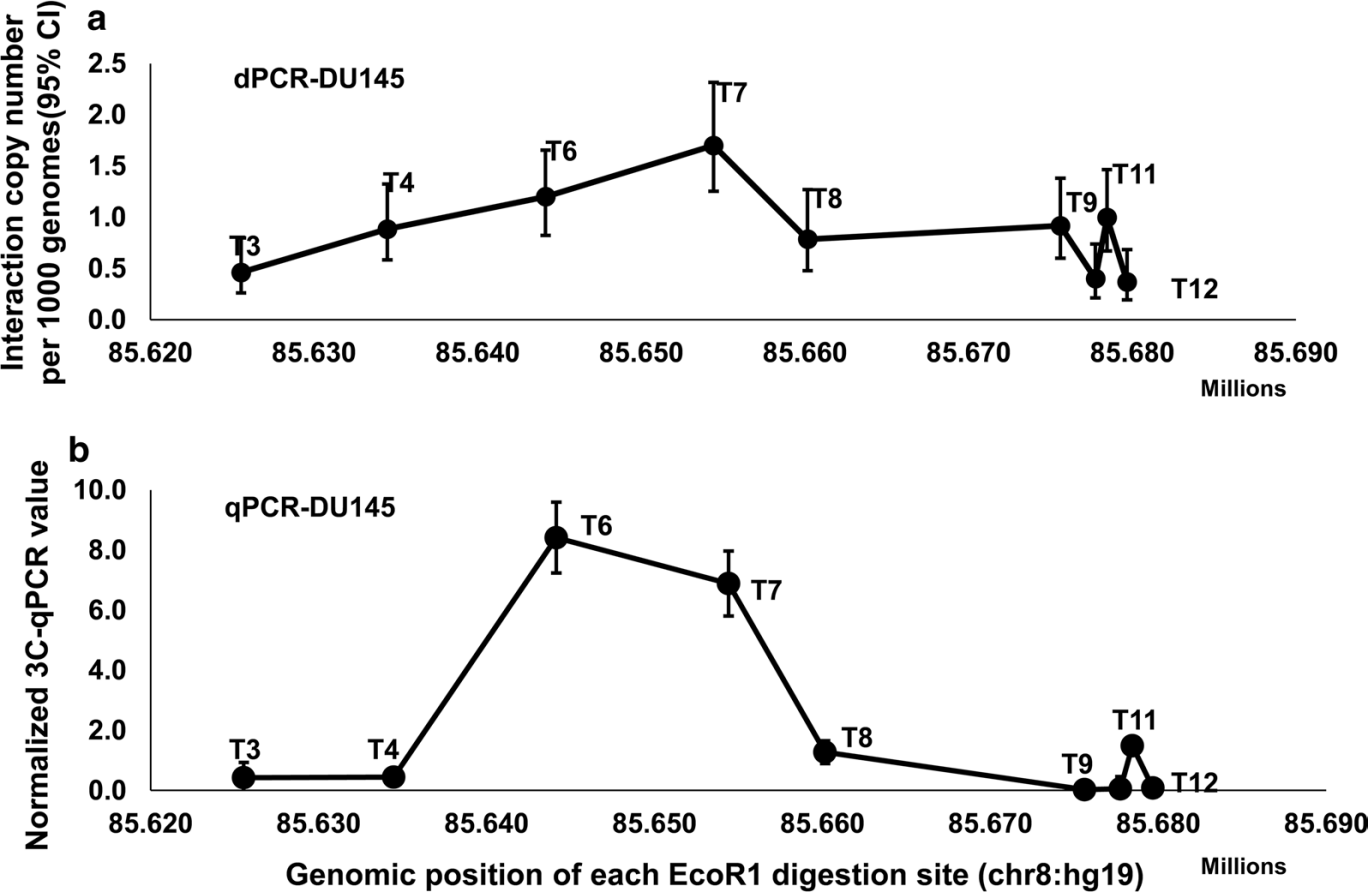
Comparison of 3C-dPCR with 3C-qPCR. **a** Copy number of ligation products at each selected restriction site detected by 3C-dPCR. Relative strong interaction signals were found with the *Eco*RI fragments covering primer T6, T7 and T11. The y-axis displays the ligation copy numbers at each *Eco*RI site per 1000 genome. The x-axis is the genomic position of each *Eco*RI site. The *error bars* represent 95% CIs. **b** 3C qPCR-based interaction signals between anchor primers and test primers. Higher interaction signals were detected with the *Eco*RI fragments covering primer T6, T7 and T11. The y-axis displays normalized 3C-qPCR values. The x-axis is the genomic position of each *Eco*RI site. *Error bars* represent standard deviation of triplicate qPCR results. The TaqMan assay at T3, T4 T8, T9, T12 fragments are beyond detection limitation of qPCR (>45Ct). Quantification values may not reflect fragment ligation frequency

To investigate the potential precision of 3C-dPCR and compare it with the established technique of 3C-qPCR, a comparison of dPCR and qPCR was performed to detect the interaction frequency between 2p11.2 and the cluster of *Eco*RI fragments on *CAPG* locus in another 3C library made from cell line DU145. The 3C product was run separately by both dPCR and standard TaqMan qPCR to directly compare the interaction frequency of different primer pairs. Figure 5a shows strong signals between the *Eco*RI fragments covering primer T7, T6, T11 and anchor primer with 1.7 (95% CI 1.26–2.32), 1.2 (95% CI 0.82–1.66) and 1.0 copy (95% CI 0.66–1.46) molecules per 1000 genomes, respectively. However, fragments near this interaction were 2–5 folds lower than the active interaction fragments. Figure 5b showed the corresponding 3C-qPCR results. Although the interaction peak was slightly different between dPCR and qPCR, the overall trend from two results were highly consistent.

## Discussion

To identify a chromatin interaction, it is necessary to demonstrate higher ligation frequency between two restriction fragments than randomly ligated fragments. Because ligation frequency is generally low between any two non-adjacent fragments [1, 5], a meaningful 3C analysis critically relies on the accurate quantification of different ligation products. In this study, we evaluated dPCR, the latest DNA quantification technology, for sensitive detection of chromatin interactions. Our results show that the 3C-dPCR is user-friendly and able to detect all previously reported interactions. Its simplicity and accuracy make it ideal for low copy number analysis such as low ligation frequency between chromatin interactions.

Currently, the most commonly used qPCR-based 3C assay has its own limitations. First, the assay requires preparation of randomly ligated control template DNA to normalize the amplification efficiency differences among different primer pairs [13]. Second, this assay generates relative quantification of ligated fragments [4, 6]. Third, the assay may not be sensitive enough to detect low frequency ligation products. Low concentration of ligation products in standard 3C library often leads to high Ct value, sometimes beyond the limitation of qPCR detection. In contrast, by sub-dividing a reaction mix into thousands of individual replicates, the dPCR assay significantly reduces the total number (hence diversity) of DNA molecules in any given partition effectively enriched for the sequences of interest and diluted out other background sequences. Therefore, dPCR assay is more sensitive and more specific than qPCR assay [14]. It also effectively overcomes qPCR biases due to primer amplification efficiency differences. In addition, the 3C-dPCR is able to generate absolute numbers of ligated target fragments and genome copies in one reaction without requirement of normalization controls. Therefore, the 3C-dPCR is simpler and more sensitive in determining low interaction frequency at the target regions of interest.

The dPCR may also simplify quality control during 3C library preparation. For example, dPCR can be used to determine efficiency of restriction enzyme digestion and proximity-based ligation. In current 3C protocol, internal control primer pair is required to accurately calculate percentage of digested fragments and ligated fragments among all available genome copies. The dPCR, however, does not have amplification bias and can accurately calculate digestion and ligation efficiency. For the low frequency ligations that are close to the lower limit of detection, dPCR system allows increasing the 3C DNA concentration in the PCR mix to provide more target ligations available for detection. The system also allows running a larger volume of the same sample on multiple chips and pooling the data into one larger “virtual” chip for low frequency ligation detection.

## Conclusion

Over the years 3C-based technologies have been evolved from single PCR assay to massive parallel sequencing assay [15–17]. Although the sequencing assays have significantly extended the scope of chromatin loop mediated long distance interaction and facilitated understanding biological mechanisms underlying gene regulation, most studies still rely on PCR-based assay to evaluate interactions at pre-defined genomic regions. By introducing dPCR into 3C assay, we show that this digital technology not only eliminates the potential variations of PCR amplification efficiency but also provides more accurate measurement of proximity-based fragment ligation frequency. The 3C-dPCR is a preferred method for sensitive and specific quantification of chromatin interactions.

## Methods

### Selection of chromatin interaction loci and primers/probes design

Previous study showed that prostate cancer risk loci at 8q24 were interacted with *MYC* region [11]. To test the feasibility of dPCR in detection of such chromatin interactions, we designed an anchor primer that was located upstream of the *Eco*RI site at chr8: 128537495 on 8q24 named as 8L. This site was shown to have an interaction peak with *MYC* in a previous report [11, 18]. Five test primers were selected downstream of each *Eco*RI site around the *MYC* gene region from chr8:128737079 to chr8:128772550 (named as *MYC*1 to *MYC*5, respectively). *MYC* gene promoter was in *MYC3* fragment (Fig. 3a). One test primer, 9 kb downstream from the anchor primer, was used as positive control (nearby ligation). Two other test primers, 85 and 92 kb away from the anchor primer were used as long-distance random ligation (negative) controls. We named the corresponding primers as 9, 85 and 92 kb accordingly. Each of these test primers was paired with the anchor primer. One pair of primer within *Eco*RI fragment (Chr8:128521424–128537496) was used to normalize genome copy number. TaqMan probes were located downstream of the anchor primer and labeled with 5′ FAM (targets) or HEX (copy number control) (Fig. 1A). The primers and probes were synthesized by Integrated DNA Technologies (Coralville, IA, USA). TaqMan probes were dissolved in TE pH 8.0 and stored at −20 °C as 2.5 μM aliquots. The sequences of the primers and probes were listed in Additional file 1: Table S1.

For the interaction between 2q11.3 and gene *CAPG*, primers and probe were designed as previously reported [12]. In brief, the anchor primer at 2q11.3 was designed near the cutting site chr2: 85778503 named 2L. Eleven test primers were spread twelve *Eco*RI cutting sites from chr2: 85619044 (T1) to chr2: 85679686 (T12), which covered the promoter and nearby region of gene *CAPG*. Adjacent ligation primer was designed on the fragment next to 2L. Each test primer was paired with the anchor primer. Self-ligation primer was designed on the same fragment with anchor primer and paired with the anchor primer to test self-ligated DNA circles. Undigested control primer was across the *Eco*RI site 2L and paired with the anchor primer (Fig. 4a). The sequences of the primers are listed in Additional file 1: Table S1.

### 3C library preparation

3C libraries were prepared as previously described [4]. Briefly, 1  ×  10^7^ cells were cross-linked with 1% formaldehyde for 10 min, and quenched with a final concentration of 0.125 mM glycine for 5 min at room temperature. Cells were counted and placed into aliquots 5 × 10^6^ cells. Each aliquot of cells was lysed with 500 μL 1× cold lysis buffer (10 Mm Tris–HCl Ph 8.0, 10 Mm NaCl, 0.2% Ige cal CA630) including 1× protease inhibitor (Roche, Indianapolis, IN, USA) for at least 15 min on ice. Cell nuclei were pelleted, washed twice with 500 μL ice cold 1× *Eco*RI buffer (NEB, Ipswich, MA, USA), and then re-suspended in 500 μL 1× * Eco*RI buffer with 0.3% SDS and incubated for 1 h at 37 °C, followed by adding 1% Triton X-100 and incubated for another 1 h to sequester the SDS. Each sample was digested overnight with 600 U restriction enzyme at 37 °C. To stop the restriction digestion, 1.6% SDS (final concentration) was added, and samples were incubated at 65  °C for 20 min. Ligation were performed at 16 °C for 4 h in 15 mL tubes containing 745 μL 10× T4 ligase Buffer, 10% Triton-X 100, 80 μL 10 mg/mL BSA, 6 mL water, 575 μL of cell lysate, 10 μL 1U/μL T4 ligase (Invitrogen, Grand Island, NY, USA). The crosslinks were reversed with Proteinase K (Invitrogen) at 65 °C overnight. 3C samples were then purified using phenol–chloroform extraction and quantified by Qubit dsDNA HS Assay (Life Technologies).

### Digital PCR

QuantStudio 3D Digital PCR System (Life Technology, Carlsbad, CA, USA) was used for the dPCR. For each Chip, reactions were performed in 18ul volume using 9 μL of 2× 3D Digital PCR master mix, 500 nM of target primer pairs, 250 nM of probes, 80 ng of 3C template DNA examining long-distance interaction copies and 8 ng of 3C samples testing the enzyme digestion efficiency and self-ligation copies. A copy number control primer/probe mix was added in the same concentration as target primer/probe mix for duplex dPCR for genome copy number determination. To exclude the false positive result caused by high level non-specific background signal from PCR amplification, ddH_2_O and random ligation control genomic LNCaP DNA after * Eco*RI digestion and T4 ligation were used as dPCR negative controls. Reaction mix was evenly loaded into a Digital PCR 20 K Chip containing 20,000 partitions. After sealing, the Chip was loaded into the Dual Flat Block GeneAmp PCR System 9700. Reactions were performed under universal cycling conditions: 96 °C for 10 min, followed by 45 cycles at 58 °C/60 °C for 2 min and 98 °C for 30 s with final extension at 60 °C for 2 min.

The Chip signal image was captured by the QuantStudio 3D Digital PCR system. Data analysis was performed using the AnalysisSuite Software (Life technology), which provided the copy number per μL reaction mix. Thresholds were determined based on results from negative control wells containing no template DNA; only wells above a minimum amplitude threshold were counted as positive. As template DNAs were randomly distributed among the all partitions, a Poisson correction was applied to correct for potential multiple copies per well. The confidence interval (CI) calculations for the absolute quantity (AQ) accounted for the Poisson error and resulted in a CI that was consistent with the random distribution of molecules across the chip, assuming that the deposition of the molecules follows a Poisson process. For the CI around the relative quantity (RQ), the absolute quantity of each target was first determined along with the CI around the AQ. The RQ was then calculated along with a CI around the RQ, consistent with the CI expected for the ratio of two types of target molecules distributed by two independent Poisson processes. For replicate chips, the combined RQ result across the replicate chips was calculated using a weighted average of the RQ result from individual chips, where the weighting factor was derived from the inverse of the CI around the RQ value from each chip [19]. The interaction frequency (=target copy number per 1000 genomes) was calculated: 1000× target copies/μL divided by genome copies/μL.

### Real-time quantitative PCR

To confirm the dPCR data, TaqMan qPCR technology was used to quantify the ligation frequency of 2p11.2 risk locus and the cluster of * Eco*RI fragments on *CAPG* locus. All PCR reactions were performed using Taqman Universal Master Mix II (Applied Biosystems, Foster City, CA, Cat# 4440038). Each 10 μL reaction consisted of 1× Taqman Universal MasterMix II, 1 μL 5uM anchor primer, 1 μL test primer, 1 μL Taqman probe (2.5 μM), and 100 ng 3C DNA. PCR cycles were as follows: an initial step 2 min at 50 °C, 10 min at 95 °C, 50 cycles of 15 s at 95 °C, 60 s at 58–60 °C. Each PCR reaction was performed in triplicate, and the data presented were the average of at least two independent experiment results for all PCR reactions. The contact frequency of each interaction pair was normalized using a 3C-control library prepared from pooled PCR products that contained 16 * Eco*RI-digested and T4 ligase-ligated fragments covering target * Eco*RI cutting sites and primer binding sites [12]. Adjacent fragment ligation frequency was used to normalize the different loading, fixation and ligation efficiencies between different cell lines.

## Additional file

**Additional file 1: Table S1.** Table of sequences of the primers and TaqMan probes used for dPCR.

## Abbreviations

3C: chromosome conformation capture
PCR: polymerase chain reaction
qPCR: quantitative PCR
dPCR: digital PCR
Ct: cycle threshold
CI: confidence interval
AQ: absolute quantity
RQ: relative quantity (RQ)
